# Online analysis of chemical composition and size distribution of fresh cigarette smoke emitted from a heated tobacco product

**DOI:** 10.1016/j.mex.2022.101912

**Published:** 2022-11-04

**Authors:** Zuoying Wen, Xiangyu Li, Xuejun Gu, Weijun Zhang, Yongqiang Pang, Xingyi Jiang, Hongwei Hou, Qingyuan Hu, Jian Wang, Long Zhang, Yong Liu, Xiaofeng Tang

**Affiliations:** a Anhui Institute of Optics and Fine Mechanics, HFIPS, Chinese Academy of Sciences, Hefei 230031, Anhui, China; b China National Tobacco Quality Supervision & Test Centre, Zhengzhou 450001, Henan, China; c Key Laboratory of Combustion and Pyrolysis, China Tobacco Anhui Industrial Co. Ltd., Hefei 230088, Anhui, China

**Keywords:** VUV photoionization mass spectrometry, Online chemical analysis, Heated tobacco product, Particulate matter, Size distribution, Cigarette smoke

## Abstract

Online analysis of chemical composition of cigarette smoke of a heated tobacco product (HTP) was performed by using a home-made vacuum ultraviolet (VUV) lamp-based photoionization time-of-flight (TOF) mass spectrometer. A capillary inlet and an aerodynamic lens were utilized to sample the gas- and particulate-phase of the HTP smoke, without dilution and pretreatment, which can be switched from each other within minutes. A thermal desorption unit was installed to vaporize the particulate-phase into gas and its vaporization temperature was determined, based on an equilibrium between the evaporation efficiency and the thermal decomposition of organic compounds. Then these species were softly ionized by VUV photons and their ions were measured by a reflectron TOF mass analyzer. Meanwhile, the puff-by-puff resolved size distributions of the HTP smoke were probed with a commercial scanning mobility particle sizers (SMPS). The mean diameters of particles firstly increase with the puff number, mainly located in the range of 200 – 300 nm, and then approached a steady state. This method was validated to measure the physical-chemical characteristics of the HTP cigarette smoke.

• A capillary inlet and an aerodynamic lens were utilized to sample the gas- and particulate-phase of the HTP smoke.
• Chemical composition of the HTP smoke was measured by using a compact VUV photoionization mass spectrometer.
• The particle size distribution of the HTP smoke without dilution was measured online.

## Graphical abstract

**Figure:**
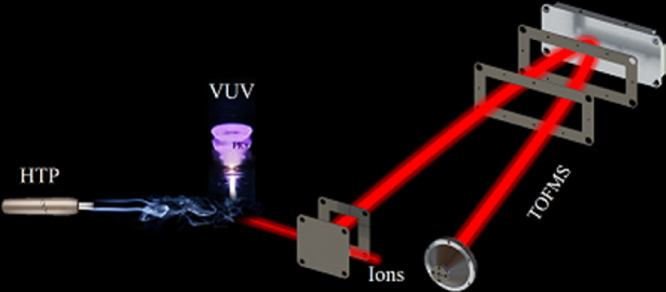

Specifications table

**Table:** 

Subject Area:	Chemistry
More specific subject area:	Analytical chemistry
Method name:	An online analytical method to analyze the physical-chemical characteristics of fresh cigarette smoke from heated tobacco product
Name and reference of original method:	*Vacuum Ultraviolet Photoionization Mass Spectrometry* *1, J. Heide et al., Nicotine & Tobacco Research, 23 (2021) 2135-2144;* *2, Y. Pan et al., Analytical Chemistry, 85 (2013) 11993-12001.*
Resource availability:	*N.A.*

## *Method details

The method developed was applied to investigate the physical and chemical characteristics of fresh cigarette smoke emitted from a heated tobacco product (HTP). The sticks of a commercial HTP from an international leading brand were used as experimental samples. A commercial linear smoking machine was automatically controlled to simulate smoking of an adult smoker and to generate cigarette aerosol under the Health Canada Intense (HCI) regime. A home-made compact vacuum ultraviolet (VUV) lamp-based photoionization time-of-flight mass spectrometer (PI-TOFMS) coupled with the smoking machine was used to online measure the gas- and particulate-phase chemical composition of the HTP smoke. In addition, the particle size distributions of the HTP smoke were measured by a commercial scanning mobility particle sizers (SMPS), which consists of a differential mobility analyzer (DMA, TSI 3081) and a condensation particle counter (CPC, TSI 3776). Using the VUV lamp-based PI-TOFMS and the SMPS coupled with the smoking machine, the time or puff-by-puff resolved information of the chemical composition and of the particle size distributions of the HTP smoke were determined. The configurations of the setup and the experimental procedures are presented below.

### Operation of the smoking machine and the samples

The commercial smoking machine consists of a cigarette holder, a magnetic three-way valve and a plunger. The smoking process was automatically controlled, and the flow rate and the time sequence of each puff were carefully calibrated. The HCI regime, that is a 55 cm^3^ per puff volume in a duration of 2 s with an interval of 30 s between puffs [1,2], was used in the experiments. A HTP stick was mounted on the cigarette holder. A Cambridge filter pad was placed or removed in the cigarette holder to separate the HTP smoke into the gas- and particulate-phase or not, dependent on the experimental conditions. The magnetic three-way valve was used to switch the flows between the cigarette holder and the plunger, for the inhalation and the exhaust of the cigarette smoke. In order to reduce the loss of volatile compounds in the smoke, the sampling tubes were used as short as possible. In addition, as the body temperature of an adult is ∼ 37°C, we did not increase the sampling temperature in the experiment and all parts of the smoking machine and the transfer tubes were kept at room temperature.

Prior to the experiments, the HTP cigarette sticks were conditioned at 22 °C with a relative humidity of 60% for at least 48 hours, according to the ISO standard of 3402 [3]. A commercial heated tobacco system was used to heat the HTP sticks. Heat was supplied to the tobacco material for a fixed period of 360 s and allowed for 12 puffs at the HCI regime. The temperature profile of the heated tobacco system was electrically controlled by its firmware and micro-controller, between 320 and 350°C [4].

### VUV lamp-based PI-TOFMS

**Fig. 1:**
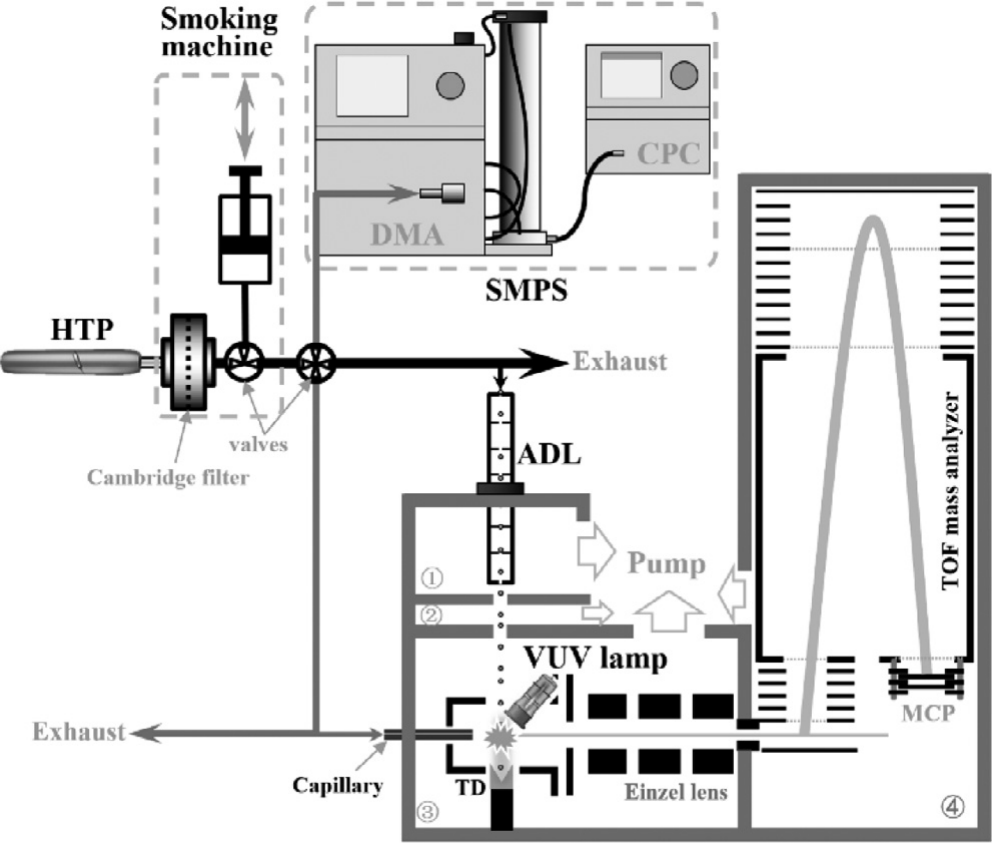
A schematic diagram of the experimental setup [5].

A schematic diagram of the experimental setup, mainly including the linear smoking machine, the SMPS and the VUV lamp-based PI-TOFMS, is presented in Fig. 1, adopted from Ref. [5]. Briefly, the smoking machine is used to generate the HTP smoke, the SMPS is adopted to measure the particle size distribution, and the mass spectrometer is employed to online analyze the gas- and particulate-phase chemical composition of the HTP smoke.

As shown in Fig. 1, the VUV lamp-based PI-TOFMS has four chambers, the source chamber ①, the differential chamber ②, the photoionization chamber ③ and the TOF chamber ④. The pressure of each chamber is maintained by their individual vacuum pumps. For example, the source chamber is evacuated by a dry scroll pump (36 m^3^/h, XDS35i, Edwards), the differential chamber, the photoionization chamber and the TOF chamber are evacuated by three turbo-molecular pumps (1260 m^3^/h, Hipace350, Pfeiffer; 1440 m^3^/h, nEXT400D, Edwards; 1080 m^3^/h, 350i, Leybold, Germany), respectively.

**Fig. 2:**
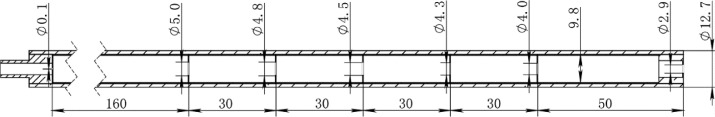
The configuration of the aerodynamic lens [5]. (Unit: mm).

The VUV lamp-based PI-TOFMS has two sampling inlets, a capillary inlet and an aerodynamic lens (ADL), which can be switched between each other within minutes. The gas-phase species are sampled via the capillary inlet with a heating temperature of 70°C, which is used to avoid condensation of volatile organic compounds (VOCs) inside. The capillary with a length of 4 cm and an inner diameter of 0.1 mm acts as an atmospheric pressure inlet to sample the gas-phase components with a flow rate of 4 cm^3^ min^−1^. The tip of capillary is inserted into the photoionization region to transfer gas-phase samples to the ionization source directly. When sampling the gas-phase species, the pressure of photoionization chamber and the TOF chamber are 1.0 × 10^−2^ and 2.4 × 10^−4^ Pa. The ADL consists of a series of lenses and spacers, as shown in Fig. 2, and is installed in the source chamber to sample and focus the particulate matters. A pinhole with 0.1 mm inner diameter is mounted on the entrance of ADL to provide a sampling flow rate of 85 cm^3^ min^−1^. After passing through the source chamber and the differential chamber, the focused particle beam arrives in the photoionization chamber, and will be vaporized into gas-phase molecules on the hot surface of a thermal desorption (TD) unit. When sampling with the ADL, the pressures of the four chambers increase at 11.6, 7 × 10^−3^, 5 × 10^−5^, and 6 × 10^−6^ Pa.

A commercial krypton discharge lamp (Heraeus) with two atomic resonant lines of krypton at hν = 10.0 and 10.6 eV is used as the VUV light source to softly ionize organic compounds at their thresholds of ionization energy. To utilize the photon flux as complete as possible, the krypton lamp is installed inside the vacuum of the photoionization chamber, right above the photoionization region, and its photon flux is ∼ 1 × 10^11^ photons s^−1^. A cage-shaped electrode with a focusing electric field inside has been developed to enhance the collection efficiency of ions [5].

The VUV light source coupled with mass spectrometry is suitable to analyze the complex organic compounds. Fragment ions can be reduced by the VUV photoionization to make the analysis of mass spectrum easier compared to other existing mass spectrometry with ’hard’ ionization method, such as electron impact ionization. Note that chemical ionization and electrospray ionization are soft ionization methods too and have been widely used in the gas-phase analysis, but they are needed to be adapted in the measurements of aerosols.

**Fig. 3:**
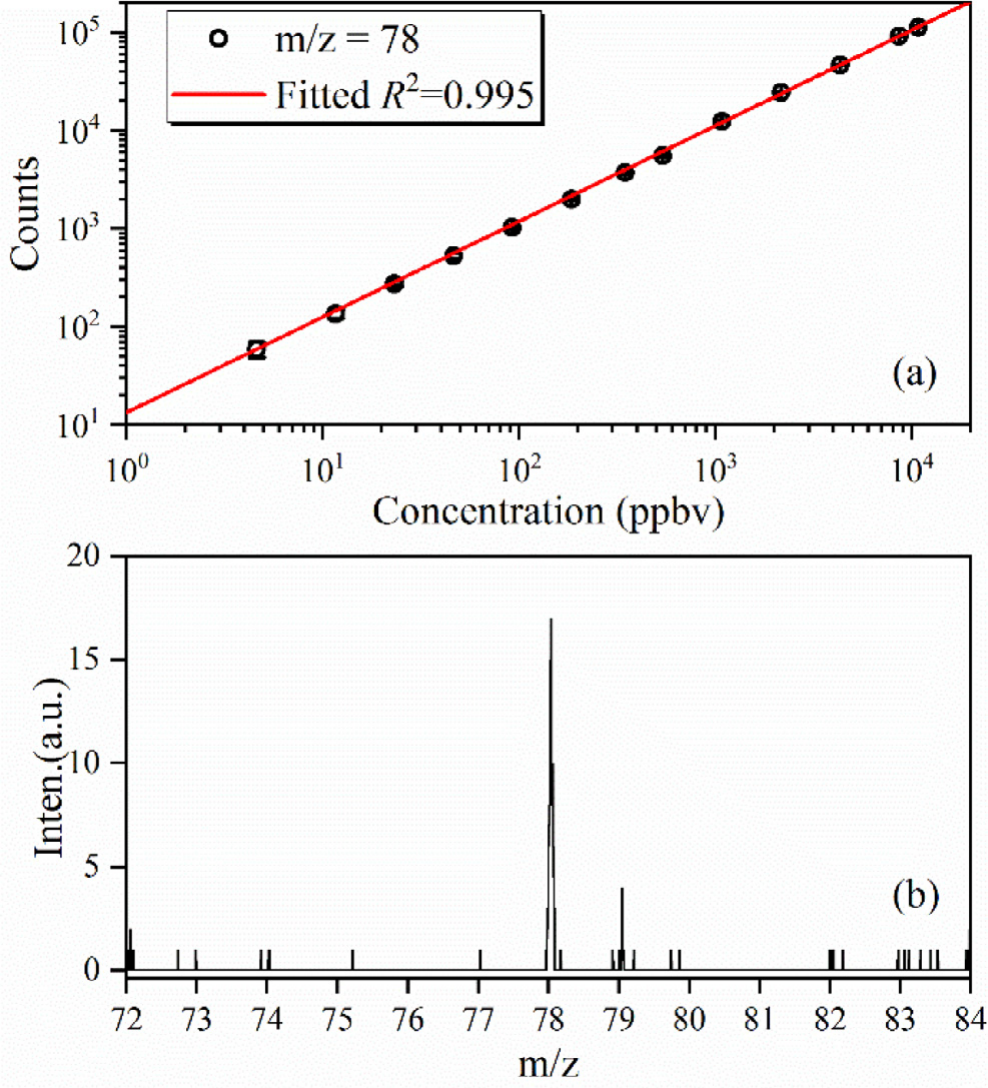
(a) The ion intensities of benzene change with its calibrated concentrations, measured with the VUV lamp-based photoionization mass spectrometer via the capillary sampling. (b) The VUV photoionization mass spectrum of benzene with a concentration of 4.5 ppbv.

An orthogonal acceleration reflectron TOF mass analyzer with a total ion flight length of ∼ 1 m is employed to analyze ion mass. The limit of detection (LOD) of the VUV lamp-based PI-TOFMS is measured with a calibrated concentration of benzene. In the experiment, the benzene gas is diluted with calibrated helium gas and the VUV photoionization mass spectra is measured with the PI-TOFMS via the capillary sampling. As shown in Fig. 3(a), the measured ion intensities of benzene change linearly with its calibrated concentrations in the range of 1-10^4^ ppbv (part per billion volume). A typical photoionization mass spectrum of benzene with a calibrated concentration of 4.5 ppbv is presented in Fig. 3(b). The peak of the ^13^C isotopic benzene (m/z = 79) can be clearly observed in the mass spectrum, providing a LOD of 0.3 ppbv. Moreover, the mass resolution of the spectrometer can be obtained from the mass peak of benzene (m/z = 78) and takes the value of M/ΔM ∼ 2000 (FWHM, the full width at half maximum).

### Mass spectral analysis

**Fig. 4:**
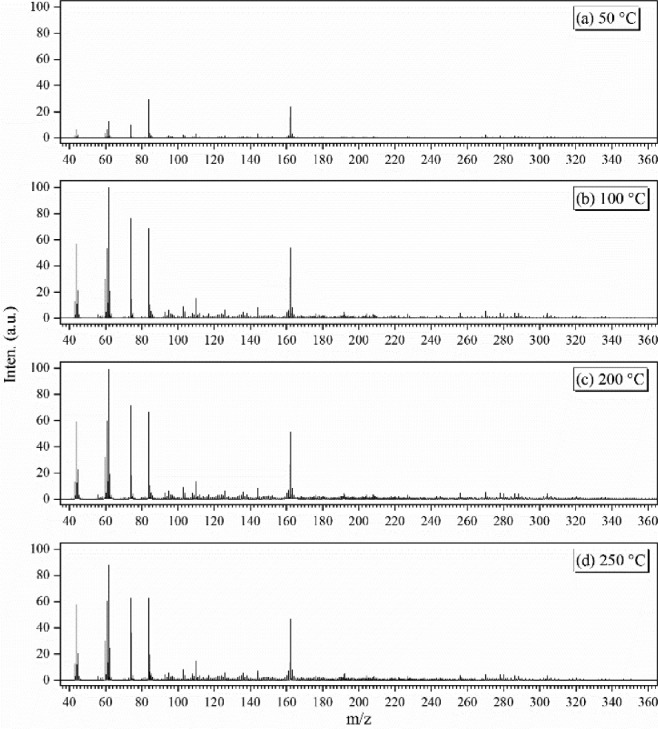
Mass spectra of HTP aerosol measured at evaporation temperature of 50, 100, 200, and 250°C.

The photoionization mass spectra of the gas-phase smoke have been reported in our recent publication [5]. In this study, we will focus on the optimization of the particulate-phase composition measurement. For example, the particulate-phase mass spectra of the HTP smoke were measured at several evaporation temperatures, and are presented in Fig. 4. Many peaks can be observed and identified in the mass spectra. The HTP cigarette smoke is an aerosol of liquid droplets and previous studies show that its major components include water, glycerol and nicotine *etc*. As shown in Fig. 4(a), the mass spectrum was measured with a TD temperature of 50°C, far below the boiling point of glycerol (290°C). But, we can see that the peaks of the fragment ions of glycerol, m/z = 43, 60, 61, 62 and 74, can still be observed in the mass spectrum. The reason is that the particles with glycerol can be easily vaporized into gas molecules and ionized in the high vacuum condition of the mass spectrometer. In addition, the molecular and fragment ions of nicotine at m/z = 84, 162 can be observed in the mass spectrum too. As the temperature of TD increased from 50°C to 100°C presented in Fig. 4(b), the intensities of glycerol and nicotine increase in the mass spectrum. Moreover, a series of other mass peaks become obvious with the TD temperature increased, *i.e.*, the peaks at m/z = 95, 103, 110, 144, as well as some organic acids with high mass. However, some ion signal intensities begin to decrease when the TD temperature exceeded 200°C, as shown in Fig. 4(c, d), due to the thermal fragmentation of vaporized molecules.

**Fig. 5:**
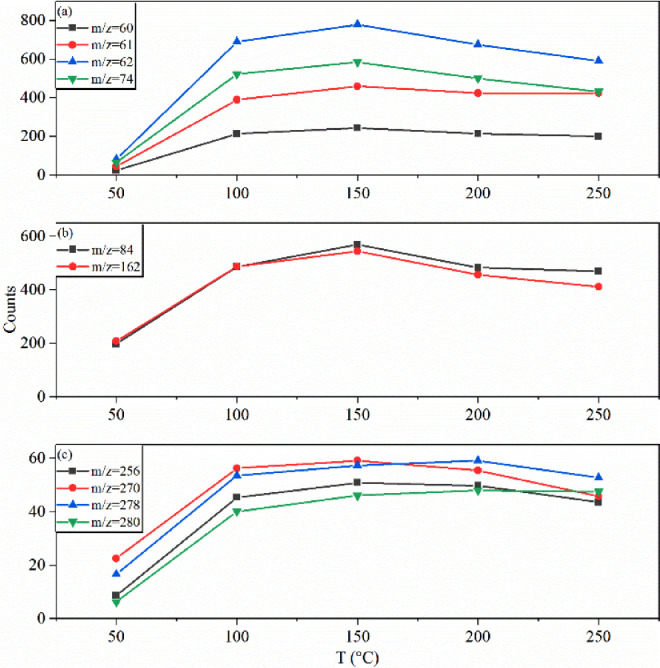
Ion signals changing with the evaporation temperatures. (a) The fragment ions of glycerol, (b) the molecular and fragment ions of nicotine, and (c) some organic acids.

The changing of the ion mass signals with the TD temperature has been presented in Fig. 5. As shown in Fig. 5(a), the intensities of the fragment ions of glycerol at m/z = 60, 61, 62, 74, change within the TD temperature of 50 – 250°C. The signals of nicotine (m/z = 84, 162) changed with TD temperature is presented in Fig. 5(b), and other mass peaks assigned as organic acids is presented in Fig. 5(c). We can see that at the beginning almost all the ion signals increase with the TD temperature. But at higher temperature, some ion signals start to decrease. So we have chosen a vaporization temperature of 200°C in our experiments. Note that some species inside the particles, such as palmitic acid and linoleic acid with their boiling point are 354.5°C and 370°C [6], might be vaporized with a less efficiency.

### Online measurement of the particle size distributions

The commercial SMPS, consisting of a DMA and a CPC, is adopted to online measure the particle size distribution of the HTP aerosol. Briefly, the HTP cigarette aerosol is firstly charged by a radioactive neutralizer (^85^Kr), the certain diameters of particles are selected by the DMA according to their electrical mobility, and the CPC located at the downstream of the DMA is used to measure the number concentration of the selected particles. Generally, the automatic scanning period of the SMPS in the size range of 10-1000 nm is 3 minutes, much longer than the smoking puff interval of 30 s, meaning that the scanning mode of the SMPS has to be changed to get the particle size distribution of cigarette smoke.

**Fig. 6:**
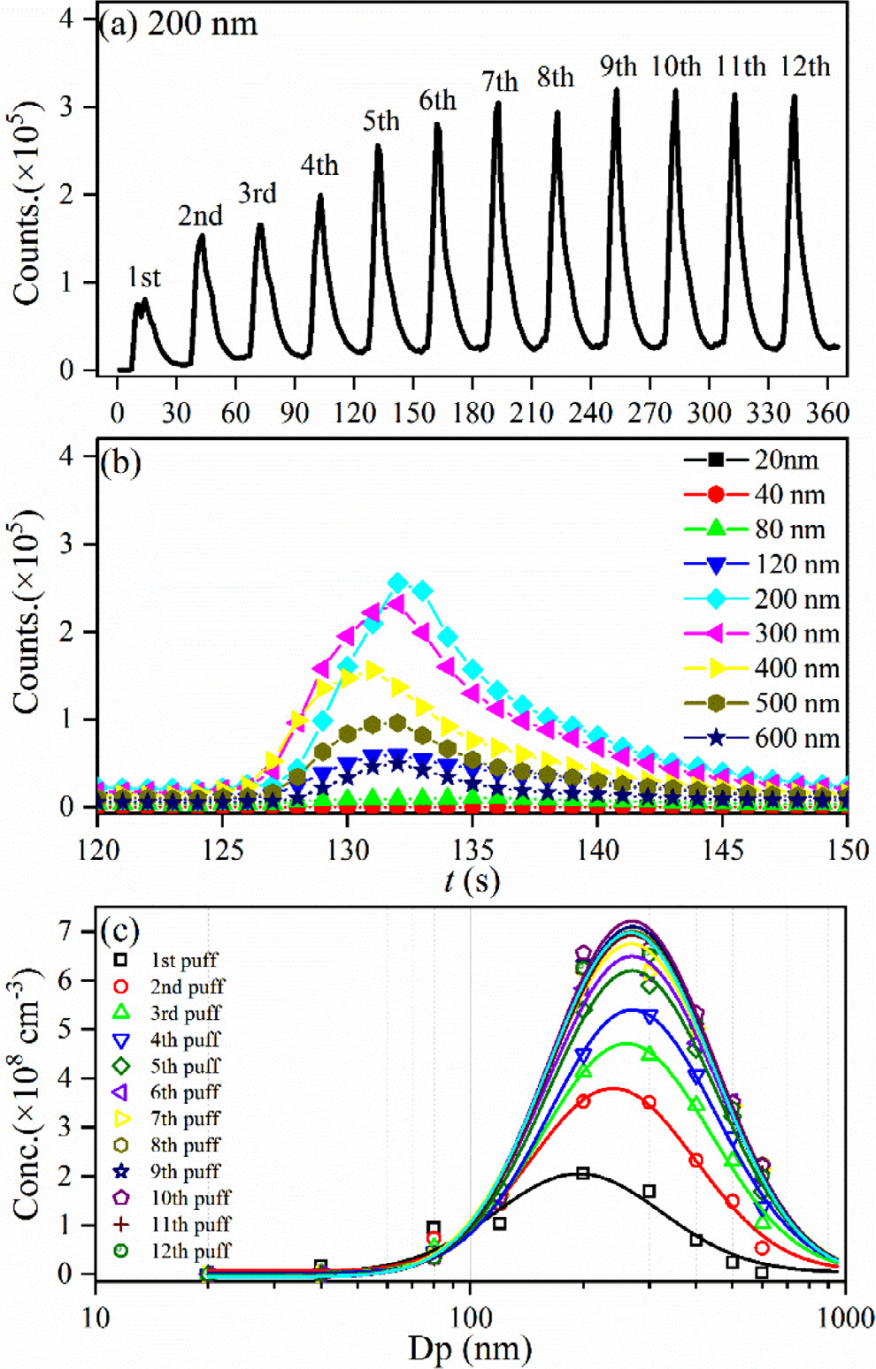
The number concentration counts of HTP aerosol measured by the SMPS. (a) The counts of HTP aerosol at 200 nm changed with puffs, (b) the counts of HTP aerosol at 20- 600 nm diameters recorded at the fifth puff, and (c) the puff-by-puff size distribution of the HTP aerosol.

For example, in the experiments, a series of certain diameters of the HTP aerosol in the range of 20-600 nm have been chosen, *i.e.*, at 20, 40, 80, 150, 200, 300, 400 and 600 nm, by adjusting the voltages of the DMA and their individual number concentration are measured by the CPC. As a representative example, the 12-puffs number concentration of the HTP aerosol at a certain diameter of 200 nm is presented in Fig. 6(a), and the fifth-puff shapes of the HTP aerosol with several certain diameters in the range of 20- 600 nm are presented in Fig. 6(b). Then the size distribution of the HTP aerosol can be obtained, as shown in Fig. 6(c), fitted with a log-normal function and their median diameters locate at 200 ∼ 300 nm with a maximal number concentration of (2 ∼ 7) × 10^8^ cm^−3^. It should be noted that at each certain diameter, we need to use a new HTP cigarette stick and its particle number concentration for the total 12 puffs can be recorded. Thus, presently we have used 9 HTP cigarette sticks to record the particle size distribution.

## Data Availability

Data will be made available on request.
